# Preventing opioid-induced nausea and vomiting: Rest your head and close your eyes?

**DOI:** 10.1371/journal.pone.0173925

**Published:** 2017-03-14

**Authors:** Fabian Heuser, Christian Schulz, Murat Sağlam, Cecilia Ramaioli, Maria Heuberger, Klaus J. Wagner, Klaus Jahn, Erich Schneider, Thomas Brandt, Stefan Glasauer, Nadine Lehnen

**Affiliations:** 1 Department of Anaesthesiology, Klinikum rechts der Isar, Technische Universität München, Munich, Germany; 2 German Centre for Vertigo and Balance Disorders, Klinikum der Universität München, Munich, Germany; 3 Department of Neurology, Klinikum der Universität München, Munich, Germany; 4 Schön Klinik, Bad Aibling, Germany; 5 Brandenburg University of Technology, Cottbus-Senftenberg, Germany; 6 Institute for Clinical Neurosciences, Klinikum der Universität München, Munich, Germany; 7 Department of Psychosomatic Medicine and Psychotherapy, Klinikum rechts der Isar, Technische Universität München, Munich, Germany; Tokai University, JAPAN

## Abstract

Although opioid-induced nausea and vomiting (OINV) is common and debilitating, its mechanism is still unclear. Recently, we suggested that opioids affect semicircular canal function and that this leads to a mismatch between canal input and other sensory information during head motion, which triggers OINV. Here, we assess if visual input is relevant for this mismatch. In a randomized-controlled crossover study 14 healthy men (26.9±3.4 years, mean±SD) were tested twice, once blindfolded and once with eyes open, with at least one-day washout. The opioid remifentanil was administered intravenously (0.15 μg/kg/min) for 60 minutes. After a thirty-minutes resting period, subjects’ head and trunk were passively moved. Nausea was rated before remifentanil start (T_0_), before the movement intervention (T_30_) and after 60 minutes (T_60_) of administration. At rest (T_0_, T_30_), median nausea ratings were zero whether subjects were blindfolded or not. Movement triggered nausea independently of visual input (nausea rating 1.5/3.0 (median/interquartile range) in the blindfolded, 2.5/6 in the eyes-open condition, χ^2^(1) = 1.3, p = 0.25). As movement exacerbates OINV independently of visual input, a clash between visual and semicircular canal information is not the relevant trigger for OINV. To prevent OINV, emphasis should be put on head-rest, eye-closure is less important.

## Introduction

Opioids are essential in the treatment of moderate to severe pain [1], but also induce debilitating nausea and vomiting. Opioid-induced nausea and vomiting (OINV) occurs in a third of all patients treated with morphine-equivalents [2] and is one of the main reasons for post-operative nausea and vomiting (PONV) [3]. PONV is a significant factor in complications such as pulmonary aspiration, dehydration, and electrolyte imbalance, delaying discharge and leading to hospital admission after ambulatory surgery, which greatly increases health care cost [4]. PONV negatively impacts patient satisfaction [5] and patients even rank it amongst the most distressing non-life-threatening side effects [1].

The exact mechanism of OINV is still not clear. Opioids affect vestibular function, as demonstrated by a decrease in caloric response with morphine [6], a diminished active vestibulo-ocular reflex (VOR) with pethidine and fentanyl administration [7], vestibular dysfunction with heroin abuse [8], and decreased semicircular canal function, measured by the VOR gain, during administration of the short-acting μ-agonist remifentanil [9,10]. Opioid receptors are present within the VOR-three-neuron arc [11,12] and in the cerebellum [13], and could mediate the changes in vestibular-ocular motor function (VOR gain). Additional oculomotor findings during opioid administration, such as gaze-evoked nystagmus, saccadic smooth pursuit and, in particular, downbeat-nystagmus, point to cerebellar involvement [7,9].

Importantly, we demonstrated that head movement greatly exacerbates nausea during administration of the opioid remifentanil, while resting protects from it [9,14]. We suggested that the change in semicircular canal input during remifentanil administration leads to a mismatch between this information and that of other sensors when the head is moved, triggering nausea and vomiting [9].

To understand the role of visual input for this mismatch and, practically, to assess whether, in addition to avoiding head movement, it makes sense to close the eyes to decrease OINV, we examined motion-dependent nausea during opioid administration with and without vision.

## Materials and methods

### Standard protocol approvals, registrations, and patient consent

Fourteen healthy men aged 26.9±3.4 years (mean±SD) gave their written consent to the study that was approved by the Ethics Committee of Technische Universität München’s Medical Faculty and performed in accordance with the Declaration of Helsinki. Subjects were free to withdraw from the experiment at any time. They were financially remunerated for taking part in the study. Subjects did not suffer from any balance disorders and were not taking any medication, explicitly no opioids.

### Opioid administration

Remifentanil, chosen because of its well-known and controllable characteristics, was administered intravenously (0.15 μg/kg/min, in analogy to Lehnen et al. [9]) during standard monitoring (ECG, non-invasive blood pressure, pulse, pulsoxymetry).

### Experimental conditions

**Fig 1:**
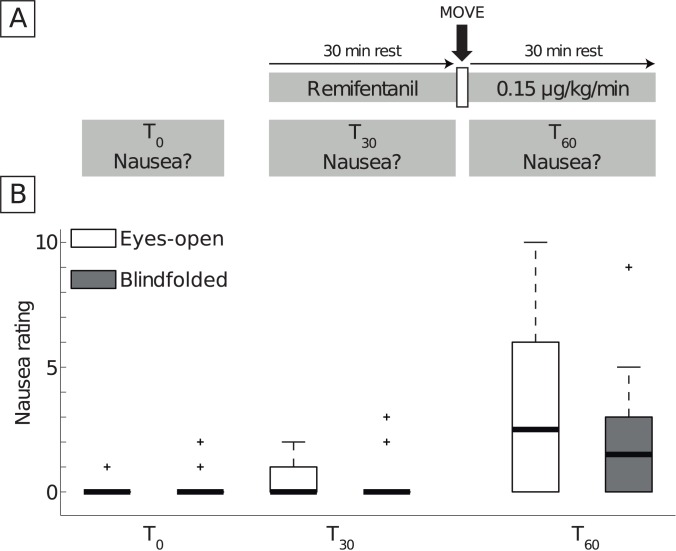
A. Experimental design (crossover study). Subjects rested in a semi-recumbent position while remifentanil was continuously administered intravenously. After 30 minutes, subjects were bent forward and backward ten times at a frequency of 1 Hz (“Move”) after which they rested again for 30 minutes. Nausea was assessed on a numerical scale from 0 (no nausea) to 10 (vomiting) before the remifentanil infusion (T_0_), during the 30 minutes of rest with remifentanil administration (T_30_), and during the 30 minutes (T_60_) after the movement intervention. The study contained two conditions: in the “blindfolded” condition subjects wore an eye patch during the entire duration of the experiment, while in the “eyes-open” condition subjects were encouraged to leave their eyes open. In a crossover design with at least one-day of washout, seven subjects were first tested with the condition “blindfolded” and then “eyes-open”, and vice versa. B. Effects of movement on nausea during remifentanil administration with and without visual input. Median (horizontal line), interquartile range (box) of nausea values quantified on a numerical scale from 0 (no nausea) to 10 (vomiting) in conditions “eyes-open” (white) and “blindfolded” (grey). Crosses designate outliers (outside 75-percentile + 1.5x interquartile range). Whiskers extend to the closest data value not considered an outlier. Data from the two subgroups of the crossover design was pooled, as there was no difference between the subgroups in all conditions (all p>0.20). During rest, nausea ratings did not differ at any time point (T_0_, T_30_ of both conditions; all medians zero, p = 0.26). Movement led to a marked increase in nausea (difference between T_0_, T_30_, and T_60_, “blindfolded”: p = 0.012, “eyes-open”: p<0.001), independently of visual input (no difference between nausea ratings at T_60_ in the "blindfolded" and "eyes-open" condition, p = 0.25)

Subjects were divided into two subgroups of equal age (26.4±3.5 and 27.3±3.6 years, mean±SD), motion-sickness-susceptibility score (assessed by the Motion Sickness Susceptibility Questionnaire, MSSQ-Short [15], 5.9±9.2 and 5.9±5.8 points) and postoperative-nausea-and-vomiting risk [3] (20±9.7% and 22±8.5%). In a crossover design, each subject was tested twice with at least one-day washout. Seven subjects were first tested “blindfolded,” then “eyes-open”, and vice-versa. In the condition “blindfolded”, subjects wore eye patches for the entire duration of the experiment. They were resting in a semi-recumbent position during 60 minutes of remifentanil administration (Fig 1A). After remifentanil had been administered for 30 minutes, subjects were bent forward and backward ten times at a frequency of 1 Hz. The condition “eyes-open” was the same as the “blindfolded” condition, but the subjects were not blinded and were encouraged to keep their eyes open.

Subjects rated nausea on a scale from zero (no nausea) to ten (vomiting) before receiving remifentanil (T_0_), after the period of 30 minutes rest (before being moved, T_30_) and after the 30 minutes following the movement intervention (T_60_). Maximal ratings for each period were analysed.

### Statistical analysis

Data were analysed offline in Matlab^®^ (Mathworks^®^, Natick, MA). Differences in nausea ratings between subgroups were assessed with an independent samples Mann-Whitney-U test (factor *order*), differences within *time* points T_0_, T_30_ and T_60_ of both *conditions* (“blindfolded”, “eyes-open”) with a related samples Friedman’s ANOVA-by-ranks (significance level p<0.05).

## Results

Nausea ratings did not differ between the subgroups for each *time* point (T_0_, T_30_, T_60_). Therefore they were independent of the *order* of the conditions (all p>0.20), and consequently, data for both subgroups of the crossover design were pooled. Fig 1B shows nausea ratings for the “eyes-open” and the “blindfolded” *conditions* at rest (T_0_, T_30_) and during the 30 minutes after the movement intervention (T_60_). At rest, nausea ratings did not differ for all *times* T_0_ and T_30_ of both *conditions* (all medians zero; χ^2^(3) = 4.0, p = 0.26). Comparison of T_0_, T_30_, and T_60_ within the conditions revealed that movement triggered nausea in both *conditions* (“blindfolded”: χ^2^(2) = 8.9, p = 0.012, “eyes-open”: χ^2^(2) = 16.7,p<0.001). Nausea ratings during T_60_ did not differ between the *conditions* (χ^2^(1) = 1.3, p = 0.25, “eyes-open” 2.5/6.0 (median/interquartile range), “blindfolded” 1.5/3.0).

## Discussion

Nausea during remifentanil administration was triggered by movement and avoided by rest in all subjects independently of visual input. This suggests that vision is not the major cause for an inter-sensory mismatch with semicircular canal input during head motion.

Remifentanil reversibly affects vestibulo-ocular reflex function, as measured by the VOR gain of the horizontal semicircular canals [9,10]. This altered information could clash with neck proprioception or other vestibular sensory information. An intra-vestibular mismatch between reduced horizontal semicircular canals [9] and not accordingly altered otolith signals seems likely. Such an intra-vestibular mismatch is acknowledged as a causative factor for seasickness (for review, see Bertolini&Straumann, 2016 [16]) and also seems to provoke space sickness where altered otolith signals in weightlessness clash with regular semicircular canal input [17].

Our study confirms the head-motion dependence of OINV with a striking median of zero on the nausea scale during rest in all conditions. As in our prior work, the incidence of OINV in our study (57/64% or 8/9 out of the 14 subjects in the “blindfolded”/“eyes-open” conditions) is higher than that predicted by the standard risk score[3] for post-operative nausea and vomiting (21±9%, mean±SD or 3/14). This is possibly because the risk score does not control for head movement, i.e., not all patients included in the calculation of the score might have experienced perioperative movement interventions similar to the ones in our study. It should be noted that there is a limitation to the interpretation of this exploratory approach due to the relatively small sample size and the fact that only men were tested.

In conclusion, our study suggests that an intra-vestibular, and not a visual-vestibular, mismatch that becomes apparent during movement exacerbates OINV. Resting thus protects from OINV, while closing the eyes appears less important. Understanding this mechanism will improve pain management with opioids. In clinical practice, especially during transient opioid application, OINV could be reduced by avoiding head movement, e.g. with a head immobilizer peri-operatively.
